# Digital patient-reported outcomes to evaluate treatment response to filgotinib in patients with ulcerative colitis

**DOI:** 10.1080/1179271X.2026.2740952

**Published:** 2026-10-12

**Authors:** Leona Mazza, Lawrence Matini, Grace Dunbar, Anita Punj Sharda, Lydia White, Andrey Kormilitzin, Hanan Azzu, Sarah Cripps, Jack Sanford, Vincent Cheung, Oliver Brain, Timothy Ambrose, Simon PL Travis, Alissa Walsh

**Affiliations:** a Faculty of Biomedical Science, Università della Svizzera Italiana, USI Lugano, Lugano, Switzerland; b Translational Gastroenterology & Liver Unit, NIHR Oxford Biomedical Research Centre, Oxford University Hospitals NHS Foundation Trust, John Radcliffe Hospital, Oxford, United Kingdom; c Department of Psychiatry, University of Oxford, Oxford, United Kingdom; d Pharmacy Department, John Radcliffe Hospital, Oxford, United Kingdom; e Kennedy Institute of Rheumatology, Translational Gastroenterology & Liver Unit and NIHR Oxford Biomedical Research Centre, University of Oxford, Oxford, United Kingdom

**Keywords:** Patient reported outcome measures, chronic disease, colitis, ulcerative, filgotinib, digital monitoring

## Abstract

**Objective:**

Patient-reported outcomes (PROs) are valuable tools for improving long-term patient outcomes and providing patient-centered care. PROs can be used to investigate real-world treatment effectiveness in patients with a chronic inflammatory disease.

**Methods:**

This was a real-world cohort study with digital patient-reported data collection by patients with ulcerative colitis (UC) using TrueColours-IBD, a web-based platform for prospective monitoring of symptoms, quality of life, and outcomes in Oxford. Effectiveness of filgotinib was compared between advanced therapy-naïve (ATN, i.e. filgotinib 1st line, *n* = 44) and advanced therapy-experienced (ATE, i.e. prior biological/small-molecule therapy, *n* = 56) patients using the following outcomes, evaluated post-induction (8–12 weeks) and at 6 and 12 months: clinical remission (Simple Clinical Colitis Activity Index (SCCAI) ≤2), clinical response (≥2 point SCCAI reduction from baseline), corticosteroid-free remission (defined as clinical remission without any prednisolone or budesonide in the 12 weeks preceding that time point), and treatment discontinuation (defined as stopped prescription of the medication).

**Results:**

There were no significant differences between the ATN and ATE groups regarding clinical remission, response, and corticosteroid-free remission at any time point, with *P* values > 0.1. More than half of the patients in each group achieved clinical remission and response at all time points. Filgotinib discontinuation rates within 12 months were comparable (*P* = 0.095).

**Conclusion:**

This study shows that PROs can serve as an important tool in evaluating real-world treatment outcomes in patients with UC using filgotinib. The results demonstrate good subjective clinical effectiveness, regardless of prior advanced therapy exposure.

## PLAIN LANGUAGE SUMMARY

This study used a digital monitoring platform called TrueColours-IBD to collect data from patients with ulcerative colitis, a chronic inflammatory disease of the bowel, to show how patient-reported data can be used to measure real-world treatment success. The platform allows patients to track and report their symptoms and subjective quality of life. These data were used to evaluate a medication called filgotinib, comparing how well it worked for patients who were completely new to so-called advanced therapies versus those who had already tried other advanced therapies before. The results show that filgotinib was effective for both groups, with no differences in treatment success between the groups. This study shows that patient-reported data are a valuable tool to evaluate treatment outcomes in patients with chronic inflammatory disease, such as ulcerative colitis.

## Introduction

Patient-reported outcomes (PROs) are clinical tools based on validated, patient-completed questionnaires and assessments.^1^ They are used to illustrate a person’s subjective view on health status, symptoms, and quality of life (QoL).^1–3^ These measures can greatly influence clinical decision-making and tailor therapies to the individual.^2^^,^^4–7^ The use of PROs has become a regulatory expectation in clinical trials, reflecting a shift towards more patient-centered research.^2^^,^^8^^,^^9^ However, assessing real-world treatment efficacy using PROs, beyond the controlled environment of randomized trials, can provide valuable data to evaluate long-term patient outcomes.^5–7^ This is particularly true for chronic illnesses, where the long-term interplay of symptoms and continuous therapies shapes patients’ QoL and subjective sense of health. The development of digital monitoring platforms, especially for chronic diseases, has been fundamental to capturing the efficacy of treatments in the real world.^10–16^

TrueColours-IBD is a web-based, self-monitoring program for patients with inflammatory bowel disease (IBD). It uses validated indices sent by email links every week for symptoms, every 2 weeks for QoL, and every 3 months for outcomes.^17^^,^^18^ Time-stamped longitudinal data are displayed on graphs, visible to patients and their clinicians. It is currently unique to Oxford, with >4500 patients with IBD enrolled, of which 2200 are diagnosed with ulcerative colitis.

Ulcerative colitis (UC) is a chronic inflammatory disease that presents with frequent and urgent bloody bowel movements and can often lead to a reduced QoL.^19^ The short- and long-term treatment goals for UC are to achieve and to maintain clinical, as well as corticosteroid-free remission, reduce mucosal inflammation, and improve the patient’s QoL.^20^ There is currently no consensus on the sequence in which available advanced therapies for UC should be prescribed, making the decision on which therapy to use first largely dependent on clinician, patient, and institutional preferences.^21^^,^^22^ JAK inhibitors are a promising alternative to biologics, with the benefits of oral administration and a rapid mechanism of action.^23^ Filgotinib, a preferential JAK1 inhibitor, has proven to be an effective drug to treat moderate-to-severe UC.^24^

Using TC-IBD, Oxford routinely collects patient-reported data in patients with UC.^17^ Consequently, there is access to prospectively collected, real-world data using validated indices, enabling the evaluation of primary outcomes based on PROs in the long term.

This real-world cohort study aims to demonstrate how the use of PROs can contribute to investigating perceived treatment effectiveness in a chronic inflammatory disease by focusing on patients with UC under filgotinib therapy as an example. Specifically, it evaluates subjective clinical outcomes and compares them between advanced therapy-naïve and advanced therapy-experienced patients.

## Methods

### Study design

This is a retrospective cohort study analyzing prospectively collected patient-reported data from patients with UC using filgotinib in Oxford. The cohort included all patients from the IBD outpatients clinic of the John Radcliffe Hospital with a diagnosis of UC (*n* = 2400) who had been prescribed filgotinib and participated in TrueColours-IBD digital monitoring (*n* = 121).^18^ Data collection was conducted between July and September 2024, according to a predetermined time-bound protocol. TrueColours-IBD is a web-based monitoring program using validated questionnaires and functioning through email prompts every week (symptoms), 2 weeks (QoL), and 3 months (outcomes). It allows PROs to be entered remotely and in real-time by the patient. All patients with UC (or Crohn’s disease) are approached to take part in TC-IBD and over 4300 (about 85% of the whole IBD cohort) do so. Patient-reported data are displayed on a personalized dashboard with graphs with red-amber-green shading for disease activity (symptoms) and QoL (Figure 1A–C). Graphs are visible to both patients and their clinicians. Ethical approval was provided through the Gastrointestinal Illness generic consent REC Ref: 21/YH/0206. Written informed consent was obtained from all participants prior to inclusion. By consenting, the participants were entering the GI Illness cohort, which is a tissue bank and database used to support research projects. All data used in this study were de-identified.

**Figure 1.**
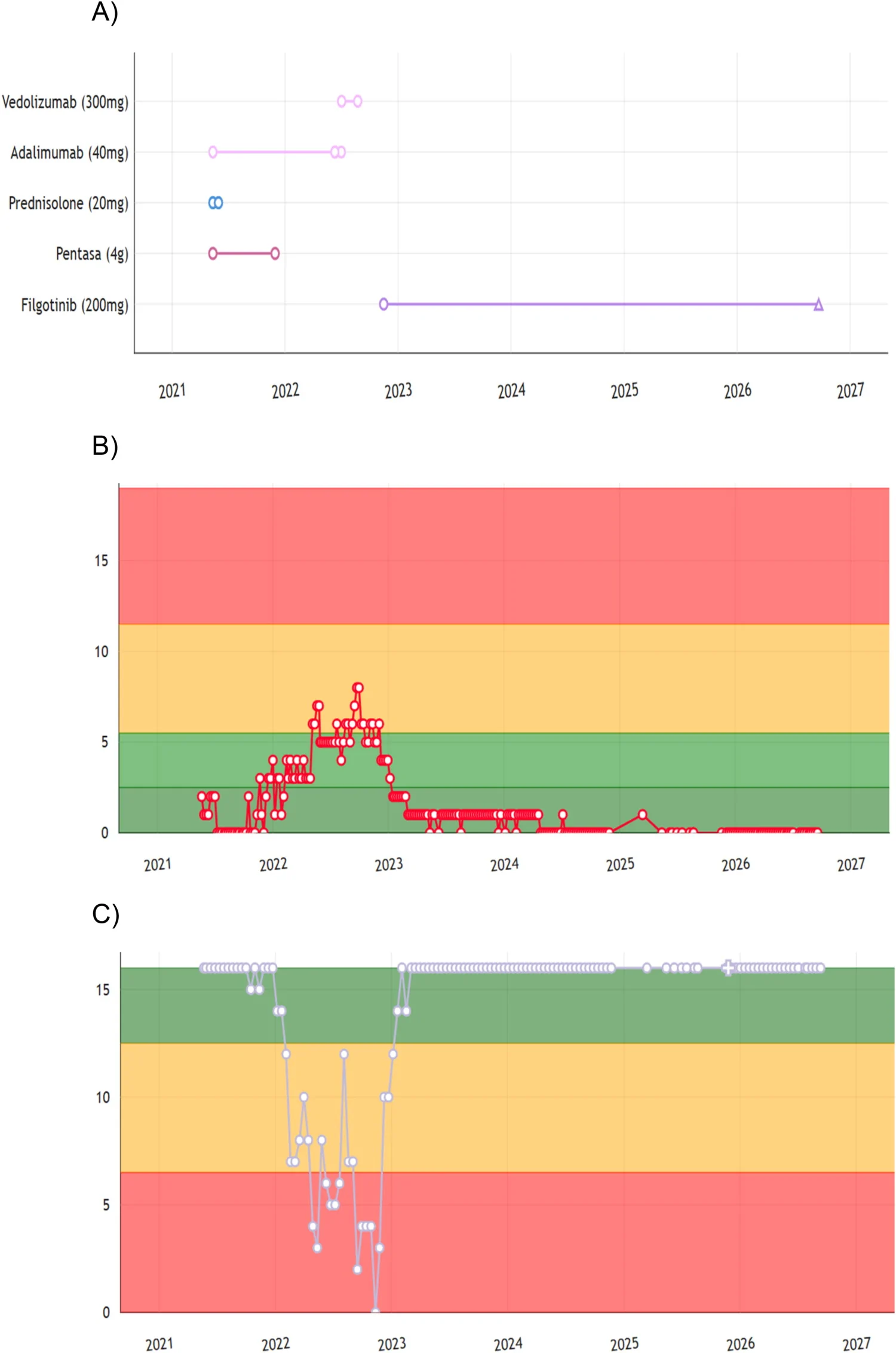
Personalized TrueColours-IBD dashboard showing (A) patient’s medication timeline, (B) disease activity (symptoms) using the Simple Clinical Colitis Activity Index, and (C) quality of life using IBD Control-8.

### Patient characteristics

Baseline characteristics were available from the TrueColours-IBD platform: age at initiation of filgotinib, gender, ethnicity, body mass index (BMI), disease duration, maximal disease extent according to the Montreal classification^25^ (E1, proctitis; E2, left-sided UC; E3, extensive UC), educational level, smoking status, extra-intestinal manifestation (EIM), and IBD-related medication history.

### Symptoms

Patient-reported data were collected through TrueColours-IBD electronic questionnaires, which included a weekly Simple Clinical Colitis Activity Index (SCCAI) for symptom assessment,^26^ and a fortnightly IBD Control-8 as a validated, disease-specific index of QoL.^27^ The SCCAI includes questions on bowel frequency (day and night), urgency of defecation, rectal bleeding, general well-being, and extraintestinal manifestations.^18^ The IBD Control-8 includes a subset of questions of the full IBD-Control questionnaire regarding disease control and QoL from the patient’s perspective.^18^^,^^27^ Questions assess perceived disease control by asking whether the patient believes their IBD is well controlled, and whether they think their current treatment is useful or needs changing. QoL is assessed by asking about missed activities because of IBD, sleep disturbances, pain and discomfort, fatigue, and anxiety or depression. The IBD Control-8 scores were color-coded as follows: red for 0–5, amber for 6–12, and green for 13–16.

Further data were collected retrospectively through medical records regarding concomitant therapy with aminosalicylates, systemic corticosteroids (prednisolone and budesonide), adverse events potentially related to filgotinib, endoscopic activity (Ulcerative Colitis Endoscopic Index of Severity (UCEIS)),^28^ and histologic activity (Nancy Index),^29^ where endoscopy had been performed as part of standard clinical care. Hemoglobin, white blood count, albumin, ferritin, transferrin, C-reactive protein (CRP), and fecal calprotectin (FCP) were recorded when they had been performed as part of standard clinical care.

### Outcomes

Patient-reported data from the TrueColours-IBD platform were collected at four time points: baseline (up to 4 weeks before initiating treatment), post-induction (8–12 weeks after initiating treatment), 6 months (21–30 weeks), and at 12 months (47–56 weeks). If time points had not been reached or filgotinib had been discontinued, the data collected up to that point were included. In case of filgotinib discontinuation, the reason for and timing of discontinuation were recorded. The mean of all TrueColours-IBD data entries for each time period was calculated.

Patients who were advanced therapy-naïve (ATN) before starting filgotinib and advanced therapy-experienced (ATE) were compared. ATN was defined as patients who had never received any advanced therapy for their UC (i.e. filgotinib 1^st^ line). ATE was defined as patients who, prior to receiving filgotinib, had already received at least one advanced therapy (meaning any licensed biological or small-molecule therapy for UC, including anti-TNF, anti-integrin, anti-IL 12/23, JAK inhibitor, or S1PR modulator therapy.^30^^,^^31^) Outcomes were defined as clinical remission (defined as an SCCAI ≤2 at the different time points), clinical response (defined as a ≥ 2 point SCCAI reduction from baseline), corticosteroid-free (CS) remission (defined as clinical remission without any prednisolone or budesonide use in the 12 weeks preceding each time point), and treatment discontinuation (defined as stopped prescription of filgotinib).^32^^,^^33^ Data used for these outcomes were strictly patient-reported and not derived from health records.

If no data entry was available at a time point, the data were specified as being missing.

### Statistical analysis

Sample size was determined by the total number of eligible patients, rather than through a power analysis, given the retrospective nature of this study. All available patient data meeting the inclusion criteria were included in the analysis to maximize statistical power. Normality of continuous variables was assessed using the Shapiro–Wilk test and visual inspection of histograms. Continuous data are reported as means and standard deviations (SD); discrete data are presented as counts and percentages. Categorical variables were analyzed using Fisher’s exact test or the Chi-Square test of Independence. Non-parametric continuous data were analyzed using the Friedman test, and if significant, a Dunn-Bonferroni post-hoc analysis was performed to identify specific differences. Additionally, a Mann-Whitney U test was conducted to investigate differences between groups. Kaplan-Meier survival analysis was performed to analyze treatment discontinuation, including the log-rank test for between-group differences. Additionally, a multivariate Cox proportional hazards regression was performed to identify predictors of filgotinib discontinuation, using univariate variables with a significance of *P* < 0.2. The P < 0.2 threshold follows established epidemiological convention for variable selection (Hosmer & Lemeshow).^34^ To identify predictors of clinical remission and clinical response post-induction, at 6 months, and 12 months, multivariate logistic regression analyses were performed, using univariate variables with a significance of *P* < 0.2. The proportional hazards assumption for the Cox model was verified using Schoenfeld residuals, and model fit for logistic regressions was assessed via Hosmer–Lemeshow goodness-of-fit tests. The mechanism of missingness was assessed using Little’s Missing Completely at Random (MCAR) test, which indicated that data were missing at random. Consequently, informative missingness was excluded, and no systematic bias was introduced into the analysis. To handle missing covariates, proportions were calculated using available data (complete cases) for descriptive analyses, whereas regression models incorporated multiple imputation. All analyses were performed with SPSS (version 29.0.1.0), and *P* values < 0.05 considered statistically significant.

## Results

### Patient and disease characteristics

Pharmacy records showed that 121 patients with UC were prescribed filgotinib, but after excluding 21 patients who declined consent and were not part of the GI Illness cohort, data were analyzed for 100 patients enrolled in TrueColours-IBD (Figure 2). All patients received filgotinib 200 mg daily, of which 44 were advanced therapy-naïve (ATN) and 56 advanced therapy-experienced (ATE). Cohort baseline and disease characteristics for ATN and ATE patients are shown in Table 1. Baseline characteristics of both cohorts were comparable, with no significant differences between the two groups. Regarding both groups (ATN and ATE), most patients were ethnically white (>90%); slightly more than half were male (61%) and never-smokers (59%).

**Figure 2.**
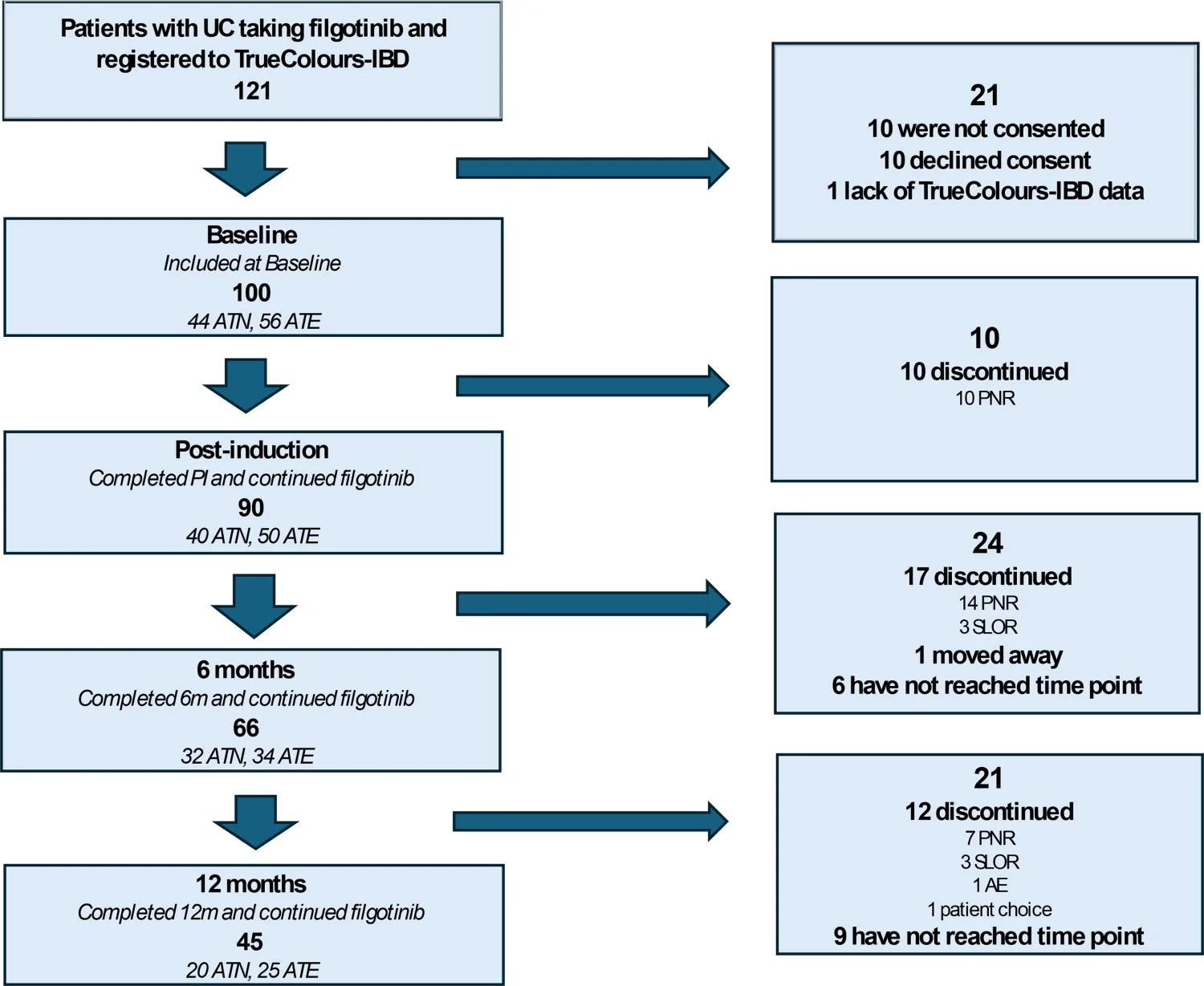
CONSORT diagram. Abbreviations: UC, ulcerative colitis; ATN, advanced therapy-naïve; ATE, advanced therapy-experienced; PNR, primary non-response; SLOR, secondary loss of response; AE, adverse events

**Table 1.** Patient and disease characteristics at baseline for the advanced therapy-naïve and advanced therapy-experienced groups.

	Advanced therapy-naïve (*n* = 44)	Advanced therapy-experienced (*n* = 56)
Age, years, mean ± SD	36.3 ± 13.6	39.4 ± 13.7
Sex, female, *n* [%]	19 [43]	19 [34]
Ethnicity, *n* [%]		
White	40 [91]	51 [91]
Asian	3 [7]	3 [5]
Mixed	0 [0]	1 [2]
Not stated	1 [2]	1 [2]
Smoking status, *n* [%]		
Former	17 [39]	20 [36]
Current	2 [5]	2 [4]
Never	25 [57]	34 [61]
BMI, kg/m², mean ± SD	26.7 ± 5.2	26.3 ± 4.6
Educational level, *n* [%]		
None	0 [0]	1 [2]
Primary	0 [0]	1 [2]
Secondary	22 [50]	23 [41]
Tertiary	22 [50]	31 [55]
Duration of UC from diagnosis, years, mean ± SD	8.5 ± 9.2	11.1 ± 8.9
Disease extent, *n* [%]		
E1	10 [23]	3 [5]
E2	23 [52]	21 [38]
E3	11 [25]	32 [57]
Extraintestinal manifestations, *n* [%]		
None	31 [71]	32 [57]
Joint	11 [25]	14 [25]
Skin	1 [2]	6 [11]
Eye	2 [5]	5 [9]
Liver/Bile duct	2 [5]	6 [11]
Concomitant therapy, *n* [%] ***		
None	15 [34]	45 [80]
5-ASA	29 [66]	11 [20]
Concomitant prednisolone use at baseline, *n* [%]	19 [43]	20 [36]
Concomitant budesonide use at baseline, *n* [%] *	20 [46]	12 [21]
SCCAI, mean ± SD	5.2 ± 2.2, (n = 28)	4.8 ± 2.5, (n = 42)
IBD control, mean ± SD	7.0 ± 4.4, (n = 23)	7.0 ± 3.4, (n = 40)
UCEIS, mean ± SD	4.2 ± 1.0, (n = 29)	4.0 ± 1.1, (n = 35)
Nancy index, mean ± SD	3.0 ± 0.9, (n = 28)	3.2 ± 0.7, (n = 34)
Prior use of		
Adalimumab, *n* [%]	–	39 [70]
Golimumab, *n* [%]	–	1 [2]
Infliximab, *n* [%]	–	10 [18]
Vedolizumab, *n* [%]	–	31 [55]
Ustekinumab, *n* [%]	–	15 [27]
Tofacitinib, *n* [%]	–	4 [7]
Ozanimod, *n* [%]	–	1 [2]
TNF antagonist and vedolizumab, *n* [%]	–	22 [39]

*P ≤ 0.05, ***P ≤ 0.001.

*Abbreviations:* BMI: body mass index; UC: ulcerative colitis; 5-ASA: 5-aminosalicylic acid; SCCAI: simple clinical colitis activity index; IBD Control: score of the IBD Control-8 questionnaire; UCEIS: ulcerative colitis endoscopic index of severity.

Disease characteristics were similar between the two groups. The only significant difference related to the use of concomitant therapies and budesonide at baseline, which were more common in the ATN (29/44 (66%) and 20/44 (46%), respectively) than in the ATE group (11/56 (20%) and 12/56 (21%), respectively). Although not statistically significant, mean disease duration was slightly shorter (8.5 years vs. 11.1 years) and mean SSCAI at baseline slightly higher (5.2 vs. 4.8) in the ATN group compared to ATE. Most patients had a disease extent of E2 in the ATN (23/44, 52%) and E3 in the ATE group (32/56, 57%). The most commonly used advanced therapy in the ATE group was adalimumab (39/56, 70%), followed by vedolizumab (31/56, 55%), and then ustekinumab (15/56, 27%) (Table 1).

### Outcomes

Clinical outcomes were analyzed using patient-reported data at four different time points: baseline, post-induction (8–12 weeks), 6 months, and 12 months. The analysis included 100 patients at baseline, and the CONSORT diagram in Figure 2 illustrates the number of discontinuations, including the reasons, over time.

Clinical remission, clinical response, and CS-free remission rates for the ATN and ATE groups are shown in Figure 3(A–C). There were no significant differences between ATN and ATE at any time point.

**Figure 3.**
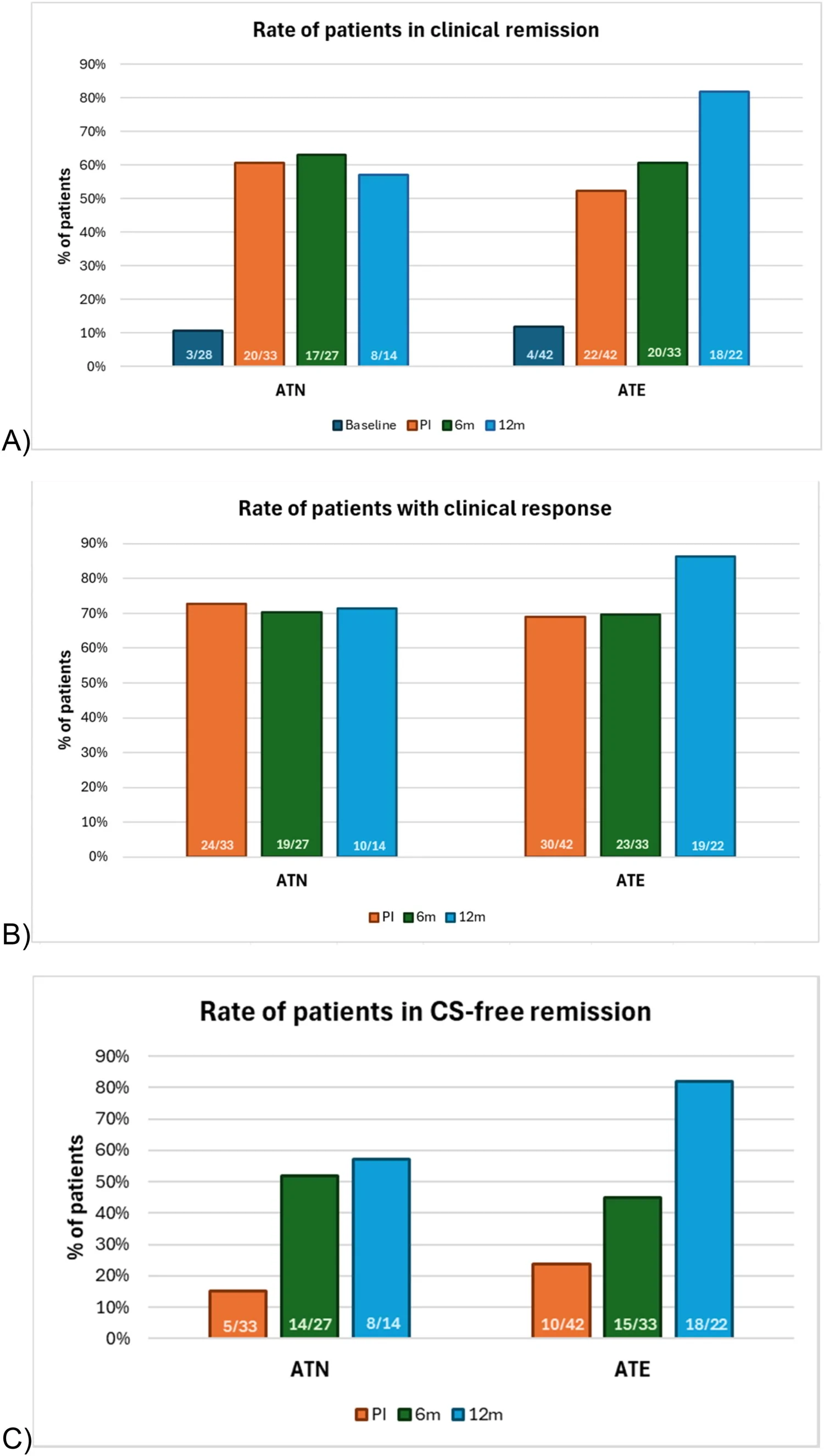
Proportions of advanced therapy-naïve and advanced therapy-experienced patients; (A) in clinical remission (SCCAI ≤ 2), (B) with clinical response (≥2 point SCCAI reduction from baseline), and (C) in CS-free remission (clinical remission without any prednisolone or budesonide use in the 12 weeks preceding that time point) at baseline, post-induction, 6 and 12 months. Abbreviations: ATN, advanced therapy-naïve; ATE, advanced therapy-experienced; PI, post-induction; SCCAI, Simple Clinical Colitis Activity Index; CS, corticosteroid

Figure 4(A) shows the SCCAI (documenting symptoms) at the four different time points for the ATN and ATE groups. In the ATN group, there was a significant difference between baseline values and the other three time points, while in the ATE group, the difference was significant between baseline, 6, and 12 months, but not for post-induction (8 weeks). There was no significant difference in SCCAI scores between the two groups at any time point.

**Figure 4 .**
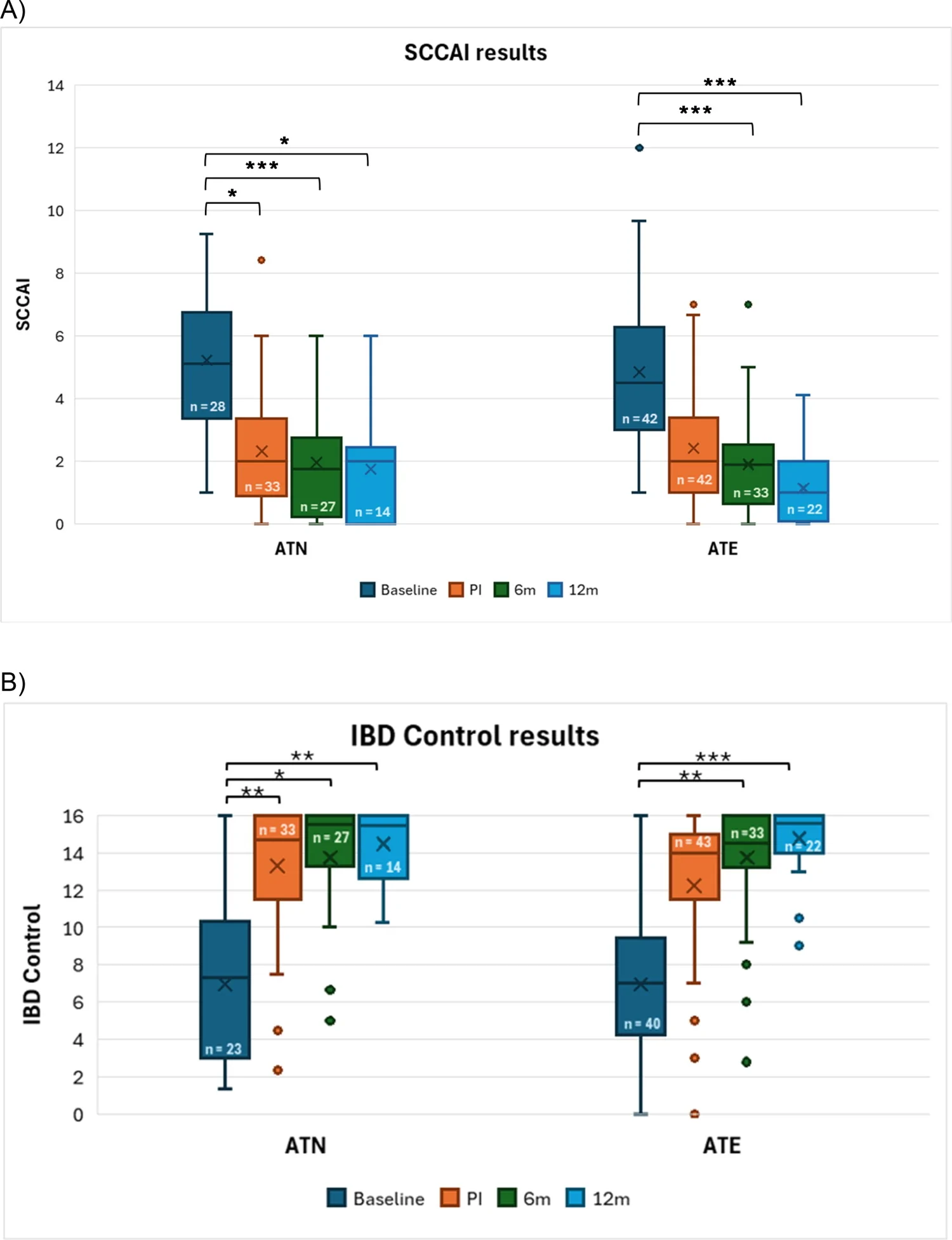
A) Simple Clinical Colitis Activity Index results of the advanced therapy-naïve and advanced therapy-experienced groups at baseline, post-induction, 6 months, and 12 months, * = P ≤ 0.05, ** = P ≤ 0.01, *** = P ≤ 0.001; B) IBD Control-8 (validated disease-specific QoL index) results of the advanced therapy-naïve and advanced therapy-experienced groups at baseline, post-induction, 6 months, and 12 months, * = P ≤ 0.05, ** = P ≤ 0.01, *** = P ≤ 0.001. Abbreviations: SCCAI, Simple Clinical Colitis Activity Index; ATN, advanced therapy-naïve; ATE, advanced therapy-experienced; PI, post-induction

Quality of life improved after treatment with filgotinib in both groups (Figure 4B). The IBD Control-8 (validated, disease-specific index for quality of life) showed a significant improvement between baseline and the other three time points in the ATN group and between baseline and 6 and 12 months in the ATE group. There were no significant differences between the two groups at any time point. The proportion of patients with IBD Control-8 scores 13–16 (positive/green), 6–12 (neutral/amber), or 0–5 (negative/red) are shown in Supplementary Figure 1, with no significant difference between the two groups.

The discontinuation rate of filgotinib was 30% in the ATN group and 46% in the ATE group (Figure 5A,B). Though the discontinuation rate was lower in the ATN group, this did not reach statistical significance (*P* = 0.095; Figure 5A).

**Figure 5 .**
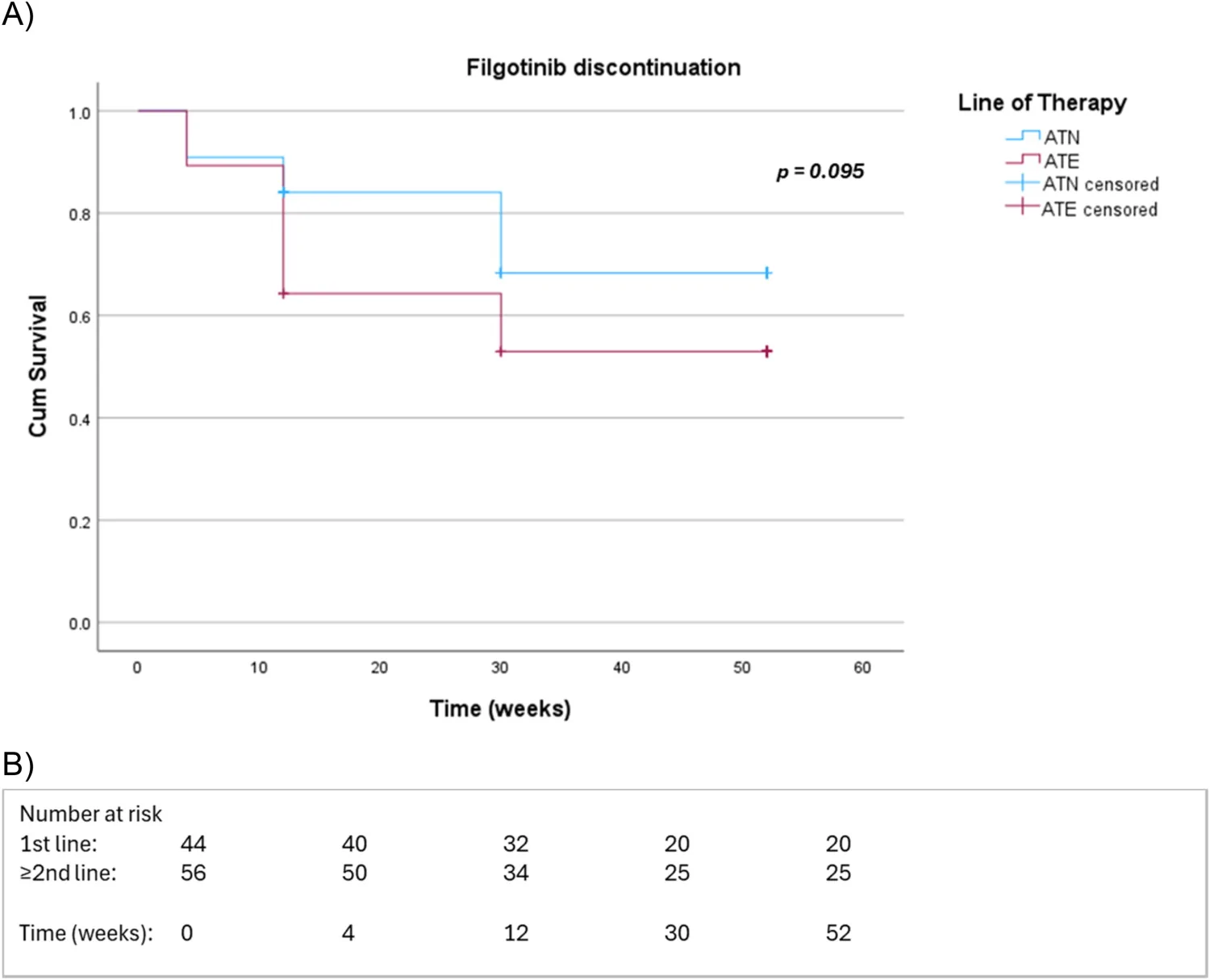
(A) Kaplan-Meier Curve showing the rate of discontinuation of filgotinib over time for the advanced therapy-naïve (blue) and advanced therapy-experienced (purple) group, *P*-value was calculated using the log-rank test; (B) number at risk for the Kaplan-Meier curve. Abbreviations: ATN, advanced therapy-naïve; ATE, advanced therapy-experienced

The most frequent reason for discontinuation was primary non-response (PNR), while others discontinued due to secondary loss of response (SLOR), adverse events, or personal choice (Figure 2). Most discontinuations occurred between post-induction and 6 months (Figure 2). When separating the cohort into the two groups, Figure 5(A,B) shows that most discontinuations occurred after 30 weeks (i.e. between 6 and 12 months) for the ATN group and between 8 weeks and 6 months for the ATE group.

### Predictors of clinical remission, clinical response, and treatment discontinuation

Multivariable analysis did not show any variables that correlated with clinical remission post-induction (Supplementary Table 1). However, multivariable analysis for clinical remission at 6 months showed a significant association with male gender (OR = 3.38, 95% CI 1.10–10.34, *P* = 0.033) (Supplementary Table 2), although this had disappeared by 12 months (Supplementary Table 3). Such a modest association is unlikely to be of clinical significance.

Multivariable analysis for predictors of clinical response at 12 months did not show any significant correlation with any of the variables (Supplementary Table 4). There was, however, a correlation between budesonide use and SCCAI at baseline and clinical response post-induction (OR = 6.72, 95% CI 1.15–39.30, *P* = 0.035, and OR = 1.57, 95% CI 1.02–2.42, *P* = 0.043, respectively) (Supplementary Table 4). Univariate analysis for predictors of clinical response at 6 months did not show any associations (Supplementary Table 4).

Regarding treatment persistence, multivariable analysis demonstrated that disease duration was inversely correlated with the likelihood of filgotinib discontinuation (OR = 0.92, 95% CI 0.85–0.99, *P* = 0.028) (Table 2, Supplementary Table 5).

**Table 2.** Multivariable analysis of variables associated with filgotinib discontinuation using cox proportional hazards regression.

Multivariable analysis of variables associated with treatment discontinuation			
Variables at baseline	Hazard ratio	95% CI	*P* value
Line of therapy			
ATN			
ATE	1.898	0.813, 4.433	0.139
Age (years)	0.998	0.971, 1.025	0.866
Disease duration	0.915	0.845, 0.991	**0.028**
Concomitant therapy			
No			
5-ASA	0.682	0.286, 1.625	0.387
Platelets	1.004	1.000, 1.008	0.067

*Note:* Bold value indicates *P* < 0.05.

*Abbreviations:* CI: confidence interval; ATN: advanced therapy-naïve; ATE: advanced therapy-experienced; 5-ASA: 5-aminosalicylic acid.

## Discussion

This study shows that PROs can be used to collect real-world data to assess treatment effectiveness and evaluate long-term outcomes in patients with IBD. It highlights the importance of patient-centered research, outside of randomized controlled trials, to enhance longitudinal care strategies, particularly in chronic diseases. Digital monitoring of PROs enables continuous monitoring between clinic visits and overcomes the limitations of clinician records, which only capture a snapshot of the disease trajectory at respective visits. This allows for a deeper understanding of the individual disease course and, consequently, a more personalized treatment perspective.^4^^,^^5^^,^^16^

Evidence in the literature suggests that continuous PRO monitoring also improves patient compliance with therapy and outcomes by enhancing patient agency and security.^10–12^^,^^14^^,^^17^ By gaining oversight of their disease trajectory, patients develop a deeper understanding of their condition, which reinforces shared decision-making with clinicians and facilitates proactivity at signs of clinical deterioration.^3^^,^^5–7^^,^^13^^,^^17^ This could prompt patients to notify health care services earlier, leading to treatment reassessment or further objective investigations, and, as a consequence, guide clinical decision-making.^17^

Despite the clear advantages of digital PRO monitoring, some difficulties remain in implementing it in routine clinical care. The reliance on patient engagement, and more importantly, digital literacy, still remains one of the biggest challenges of digital PROs.^3^ Additionally, reviewing and addressing PRO data during clinic visits may be perceived as an increased workload, making the integration of digital PROs into routine workflows more difficult.^3^^,^^17^

These real-world data are a practical example for assessing treatment outcomes through PROs. Though the efficacy of filgotinib has already been demonstrated in randomized controlled trials, our PROs-based real-world data also show that filgotinib is effective at inducing and maintaining clinical remission, clinical response, and CS-free remission, regardless of previous advanced therapy exposure.^35^ The more frequent budesonide use at baseline in the ATN group and its independent correlation with clinical response post-induction could, however, be a potential confounder and mask differences between the two groups. These results should therefore be interpreted with caution.

Similar results were reported in a Japanese multicenter retrospective study (*n* = 238) analyzing real-world data on the effectiveness and safety of filgotinib and comparing the former between ATN and ATE patient cohorts.^36^ This study had a comparable study design, looking at clinical remission, clinical response, and CS-free remission at weeks 10, 26, and 58, and it found no significant differences between the ATN and ATE groups concerning the outcomes at the different time points. However, they identified the number of prior advanced therapies to be inversely associated with clinical remission, which we did not find in our multivariable analysis. This discrepancy may reflect differences in covariate adjustment and statistical power.

In contrast to our data, a retrospective observational study of 286 patients from multiple UK centers showed significantly better clinical remission and response rates in the ATN group than in the ATE group.^37^ It should be noted that the UK study only analyzed patients who met specific criteria for disease activity (SCCAI ≥3 or Partial Mayo Score ≥ 2 and UCEIS ≥ 2 or Mayo Endoscopic Score ≥ 1) at initiation, where we included all patients where a clinical decision had been made to start filgotinib. This meant that the UK study had a higher disease burden at baseline compared to our cohort, which in UC is known to be associated with a higher rate of treatment failure.^38^ This discrepancy could, however, also stem from our smaller sample size.

Combining data from medical records, TC-IBD, and PROs can provide a valuable tool to understand individual disease courses and potentially guide clinical decision-making. Our analysis shows an exact overlap of patients who had clinical remission and CS-free remission at 12 months for both groups, suggesting that filgotinib promotes CS-free remission and reduces corticosteroid use long-term. Comparable results were reported in a post-hoc analysis of the SELECTION study, where over 90% of patients in remission at week 58 were CS-free for the last 6 months.^39^

Though the use and importance of PROs is increasing in real-world studies and randomized controlled trials regarding IBD, many commonly used measures do not align with regulatory recommendations or remain unvalidated.^40–42^ Further studies are required to develop and validate new patient-reported outcome measures with standardized outcome definitions, specifically for IBD, to provide a foundation for their use in future research.^40^

The study has limitations, as well as several strengths that should be acknowledged. Limitations of this study include the fact that this was a retrospective analysis, where selection bias for prescribing filgotinib and confounders could not be fully excluded. As data collection of PROs depends on patient compliance with how often patients answer the questionnaires, data availability varies for each time point. Consequently, missing data were frequent in several variables, resulting in small sample sizes and reduced statistical power of these analyses, especially for the outcomes at 12 months. This highlights the challenge of digital PROs for substantial data collection. Incentives to avoid this include the use of digital prompts (such as email or text), visual images of the outcomes of data collection (such as the red-amber-green graphs), and (most of all) the interest of health care professionals in the PRO data. This simply means examining the record with the patient or commenting on it at the clinic visit.^17^ Additionally, this study was limited by a substantial amount of missing data regarding objective markers of inflammation (biochemical, fecal, endoscopic, or histological), as they were only collected when available as part of routine clinical care. Consequently, clinical effectiveness was assessed exclusively via PROs and could not be validated against objective markers of inflammation and disease activity. Notable strengths of this study include real-world data from digital and prospectively collected patient-reported outcomes, which allow the analysis of the effectiveness of filgotinib by line of therapy at different time points.

This cohort study shows that PROs can be used to evaluate treatment effectiveness, outside of randomized controlled trials. The results demonstrate good subjective clinical effectiveness and outcomes in patients with UC treated with filgotinib, regardless of prior advanced therapy exposure. The message is clear: Digital PROs can serve as an important tool in evaluating long-term outcomes in patients with chronic inflammatory disease such as UC and thus, have unexploited potential in guiding clinical care.

## Supplementary Material

Supplemental online material PROM Journal.docx

## Data Availability

The data used in this work were generated as part of routine care at the John Radcliffe Hospital. The data underlying this article will be shared on reasonable request to the corresponding author.
