# Supplementary material for: Digital patient-reported outcomes to evaluate treatment response to filgotinib in patients with ulcerative colitis
Source: Patient Relat Outcome Meas. 2026 Oct 12;17(1):149–61. doi: 10.1080/1179271X.2026.2740952 (PMC13671468; doi:10.1080/1179271X.2026.2740952)
Supplement: Supplementary file 1 [file DPRO_A_2740952_SM7966.docx]

**Supplementary materials**

Supplementary Table 1: Univariate and Multivariable Analysis of Variables associated with Clinical Remission (SCCAI ≤ 2) post-Induction using Logistic Regression

| Univariate and Multivariable analysis of variables associated with remission post-induction | | | | | | | | |
| --- | --- | --- | --- | --- | --- | --- | --- | --- |
|  | Univariate | | | | Multivariable | | | |
| Variables at baseline | n | Odds ratio | 95% CI | ***P* value** | n | Odds ratio | 95% CI | ***P* value** |
| Line of therapy | 75 |  |  |  |  |  |  |  |
| ATN |  |  |  |  |  |  |  |  |
| ATE |  | 0.725 | 0.284, 1.802 | 0.477 |  |  |  |  |
| Age (years) | 75 | 0.997 | 0.965, 1.030 | 0.866 |  |  |  |  |
| Sex | 75 |  |  |  |  |  |  |  |
| Female |  |  |  |  |  |  |  |  |
| Male |  | 0.982 | 0.391, 2.470 | 0.970 |  |  |  |  |
| BMI | 75 | 0.946 | 0.861, 1.039 | 0.242 |  |  |  |  |
| Disease duration | 75 | 1.004 | 0.952, 1.058 | 0.880 |  |  |  |  |
| Disease extent | 75 |  |  |  |  |  |  |  |
| E1 |  |  |  |  |  |  |  |  |
| E2 |  | 0.769 | 0.177, 3.336 | 0.726 |  |  |  |  |
| E3 |  | 0.875 | 0.211, 3.629 | 0.854 |  |  |  |  |
| Smoker | 75 |  |  |  |  |  |  |  |
| Never |  |  |  |  |  |  |  |  |
| Former |  | 1.105 | 0.418, 2.923 | 0.840 |  |  |  |  |
| Current |  | 0.253 | 0.024, 2.631 | 0.250 |  |  |  |  |
| EIM | 75 |  |  |  |  |  |  |  |
| No |  |  |  |  |  |  |  |  |
| Yes |  | 1.312 | 0.515, 3.347 | 0.569 |  |  |  |  |
| Concomitant therapy | 75 |  |  |  | 72 |  |  |  |
| No |  |  |  |  |  |  |  |  |
| 5-ASA |  | 2.530 | 0.971, 6.595 | **0.058** |  | 2.435 | 0.802, 7.396 | 0.116 |
| Prednisolone use | 75 |  |  |  | 72 |  |  |  |
| No |  |  |  |  |  |  |  |  |
| Yes |  | 0.376 | 0.145, 0.979 | **0.045** |  | 0.619 | 0.200, 1.913 | 0.405 |
| Budesonide use | 75 |  |  |  | 72 |  |  |  |
| No |  |  |  |  |  |  |  |  |
| Yes |  | 4.200 | 1.356, 13.014 | **0.013** |  | 3.478 | 0.981, 12.331 | 0.054 |
| SCCAI | 72 | 0.982 | 0.781, 1.234 | 0.873 |  |  |  |  |
| Nancy Index | 72 | 0.864 | 0.491, 1.518 | 0.61 |  |  |  |  |
| Platelets | 72 | 0.993 | 0.984, 1.001 | **0.078** | 72 | 0.993 | 0.984, 1.001 | 0.090 |
| Ferritin | 72 | 1.001 | 0.998, 1.004 | 0.615 |  |  |  |  |

Note: Bold values indicate P < 0.05

*Abbreviations: SCCAI, simple clinical colitis activity index; CI, confidence interval; ATN, advanced therapy-naïve; ATE, advanced therapy-experienced; BMI, body mass index; EIM, extraintestinal manifestation; 5-ASA, 5-aminosalicylic acid*

Supplementary Table 2: Univariate and Multivariable Analysis of Variables associated with Remission (SCCAI ≤ 2) at 6 Months using Logistic Regression

| Univariate and Multivariable analysis of variables associated with remission at 6 months | | | | | | | | |
| --- | --- | --- | --- | --- | --- | --- | --- | --- |
|  | Univariate | | | | Multivariable | | | |
| Variables at baseline | n | Odds ratio | 95% CI | ***P* value** | n | Odds ratio | 95% CI | ***P* value** |
| Line of Therapy | 60 |  |  |  |  |  |  |  |
| ATN |  |  |  |  |  |  |  |  |
| ATE |  | 0.905 | 0.317, 2.580 | 0.852 |  |  |  |  |
| Age (years) | 60 | 1.005 | 0.969, 1.043 | 0.772 |  |  |  |  |
| Sex | 60 |  |  |  | 60 |  |  |  |
| Female |  |  |  |  |  |  |  |  |
| Male |  | 3.241 | 1.096, 9.581 | **0.034** |  | 3.375 | 1.102, 10.342 | **0.033** |
| BMI | 60 | 0.941 | 0.841, 1.053 | 0.286 |  |  |  |  |
| Disease duration | 60 | 1.027 | 0.962, 1.096 | 0.425 |  |  |  |  |
| Disease extent | 60 |  |  |  |  |  |  |  |
| E1 |  |  |  |  |  |  |  |  |
| E2 |  | 1.250 | 0.271, 5.765 | 0.775 |  |  |  |  |
| E3 |  | 0.970 | 0.221, 4.259 | 0.967 |  |  |  |  |
| Smoker | 60 |  |  |  | 60 |  |  |  |
| Never |  |  |  |  |  |  |  |  |
| Former |  | 1.050 | 0.335, 3.292 | 0.934 |  | 1.218 | 0.366, 4.048 | 0.748 |
| Current |  | 0.188 | 0.018, 2.002 | **0.166** |  | 0.188 | 0.016, 2.171 | 0.181 |
| EIM | 60 |  |  |  |  |  |  |  |
| No |  |  |  |  |  |  |  |  |
| Yes |  | 0.558 | 0.194, 1.601 | 0.278 |  |  |  |  |
| Concomitant therapy | 60 |  |  |  |  |  |  |  |
| No |  |  |  |  |  |  |  |  |
| 5-ASA |  | 1.830 | 0.635, 5.271 | 0.263 |  |  |  |  |
| Prednisolone use | 60 |  |  |  |  |  |  |  |
| No |  |  |  |  |  |  |  |  |
| Yes |  | 1.061 | 0.366, 3.073 | 0.914 |  |  |  |  |
| Budesonide use | 60 |  |  |  |  |  |  |  |
| No |  |  |  |  |  |  |  |  |
| Yes |  | 1.535 | 0.486, 4.845 | 0.465 |  |  |  |  |
| SCCAI | 57 | 0.955 | 0.727, 1.255 | 0.741 |  |  |  |  |
| Nancy Index | 57 | 0.940 | 0.452, 1.955 | 0.867 |  |  |  |  |
| Platelets | 57 | 1.002 | 0.995, 1.010 | 0.571 |  |  |  |  |
| Ferritin | 57 | 1.001 | 0.996, 1.006 | 0.754 |  |  |  |  |

Note: Bold values indicate P < 0.05

*Abbreviations: SCCAI, simple clinical colitis activity index; CI, confidence interval; ATN, advanced therapy-naïve; ATE, advanced therapy-experienced; BMI, body mass index; EIM, extraintestinal manifestation; 5-ASA, 5-aminosalicylic acid*

Supplementary Table 3: Univariate and Multivariable Analysis of Variables associated with Remission (SCCAI ≤ 2) at 12 Months using Logistic Regression

| Univariate and Multivariable analysis of variables associated with remission at 12 months | | | | | | | | |
| --- | --- | --- | --- | --- | --- | --- | --- | --- |
|  | Univariate | | | | Multivariable | | | |
| Variables at baseline | n | Odds ratio | 95% CI | ***P* value** | n | Odds ratio | 95% CI | ***P* value** |
| Line of therapy | 36 |  |  |  | 36 |  |  |  |
| ATN |  |  |  |  |  |  |  |  |
| ATE |  | 3.375 | 0.742, 15.349 | **0.115** |  | 1.768 | 0.152, 20.569 | 0.646 |
| Age (years) | 36 | 1.024 | 0.974, 1.076 | 0.349 |  |  |  |  |
| Sex | 36 |  |  |  |  |  |  |  |
| Female |  |  |  |  |  |  |  |  |
| Male |  | 1.067 | 0.240, 4.740 | 0.932 |  |  |  |  |
| BMI | 36 | 0.896 | 0.779, 1.031 | **0.125** | 36 | 0.939 | 0.793, 1.111 | 0.462 |
| Disease duration | 36 | 1.036 | 0.933, 1.150 | 0.506 |  |  |  |  |
| Disease extent | 36 |  |  |  |  |  |  |  |
| E1 |  |  |  |  |  |  |  |  |
| E2 |  | 1.467 | 0.184, 11.718 | 0.718 |  |  |  |  |
| E3 |  | 2.667 | 0.298, 23.858 | 0.380 |  |  |  |  |
| Smoker | 36 |  |  |  |  |  |  |  |
| Never |  |  |  |  |  |  |  |  |
| Former |  | 0.982 | 0.212, 4.553 | 0.982 |  |  |  |  |
| Current |  | 0.357 | 0.019, 6.850 | 0.495 |  |  |  |  |
| EIM | 36 |  |  |  |  |  |  |  |
| No |  |  |  |  |  |  |  |  |
| Yes |  | 1.500 | 0.341, 6.592 | 0.591 |  |  |  |  |
| Concomitant therapy | 36 |  |  |  | 36 |  |  |  |
| No |  |  |  |  |  |  |  |  |
| 5-ASA |  | 0.156 | 0.027, 0.890 | **0.036** |  | 0.283 | 0.027, 3.001 | 0.294 |
| Prednisolone use | 36 |  |  |  |  |  |  |  |
| No |  |  |  |  |  |  |  |  |
| Yes |  | 0.733 | 0.170, 3.169 | 0.678 |  |  |  |  |
| Budesonide use | 36 |  |  |  |  |  |  |  |
| No |  |  |  |  |  |  |  |  |
| Yes |  | 0.860 | 0.172, 4.285 | 0.854 |  |  |  |  |
| SCCAI | 36 | 1.147 | 0.633, 2.077 | 0.631 |  |  |  |  |
| Nancy Index | 36 | 2.934 | 0.979, 8.793 | **0.055** | 36 | 3.509 | 0.674, 18.277 | 0.132 |
| Platelets | 36 | 1.001 | 0.992, 1.010 | 0.854 |  |  |  |  |
| Ferritin | 36 | 1.001 | 0.996, 1.006 | 0.643 |  |  |  |  |

Note: Bold values indicate P < 0.05

*Abbreviations: SCCAI, simple clinical colitis activity index; CI, confidence interval; ATN, advanced therapy-naïve; ATE, advanced therapy-experienced; BMI, body mass index; EIM, extraintestinal manifestation; 5-ASA, 5-aminosalicylic acid*

Supplementary Table 4: Univariate and Multivariable Analysis of Variables associated with Response (≥2 point SCCAI reduction from baseline) Post-Induction, at 6 Months, and at 12 Months using Logistic Regression

| Univariate and Multivariable analysis of variables associated with response post-induction | | | | | | | | |
| --- | --- | --- | --- | --- | --- | --- | --- | --- |
|  | Univariate | | | | Multivariable | | | |
| Variables at baseline | n | Odds ratio | 95% CI | ***P* value** | n | Odds ratio | 95% CI | ***P* value** |
| Line of therapy | 75 |  |  |  |  |  |  |  |
| ATN |  |  |  |  |  |  |  |  |
| ATE |  | 0.837 | 0.305, 2.291 | 0.728 |  |  |  |  |
| Age (years) | 75 | 0.989 | 0.954, 1.025 | 0.543 |  |  |  |  |
| Sex | 75 |  |  |  | 72 |  |  |  |
| Female |  |  |  |  |  |  |  |  |
| Male |  | 0.389 | 0.132, 1.149 | **0.088** |  | 0.678 | 0.173, 2.661 | 0.577 |
| BMI | 75 | 1.040 | 0.937, 1.155 | 0.464 |  |  |  |  |
| Disease duration | 75 | 0.978 | 0.926, 1.032 | 0.417 |  |  |  |  |
| Disease extent | 75 |  |  |  |  |  |  |  |
| E1 |  |  |  |  |  |  |  |  |
| E2 |  | 0.450 | 0.080, 2.542 | 0.366 |  |  |  |  |
| E3 |  | 0.675 | 0.122, 3.736 | 0.653 |  |  |  |  |
| Smoker | 75 |  |  |  | 72 |  |  |  |
| Never |  |  |  |  |  |  |  |  |
| Former |  | 2.276 | 0.718, 7.216 | **0.162** |  | 1.499 | 0.367, 6.123 | 0.573 |
| Current |  | 0.517 | 0.066, 4.045 | 0.530 |  | 0.182 | 0.011, 2.974 | 0.231 |
| EIM | 75 |  |  |  |  |  |  |  |
| No |  |  |  |  |  |  |  |  |
| Yes |  | 1.643 | 0.576, 4.689 | 0.354 |  |  |  |  |
| Concomitant therapy | 75 |  |  |  |  |  |  |  |
| No |  |  |  |  |  |  |  |  |
| 5-ASA |  | 1.448 | 0.521, 4.030 | 0.478 |  |  |  |  |
| Prednisone use | 75 |  |  |  | 72 |  |  |  |
| No |  |  |  |  |  |  |  |  |
| Yes |  | 0.514 | 0.187, 1.413 | **0.197** |  | 0.399 | 0.078, 2.050 | 0.270 |
| Budesonide use | 75 |  |  |  | 72 |  |  |  |
| No |  |  |  |  |  |  |  |  |
| Yes |  | 6.562 | 1.387, 31,052 | **0.018** |  | 6.719 | 1.148, 39.303 | **0.035** |
| SCCAI | 72 | 1.398 | 1.008, 1.938 | **0.045** | 72 | 1.568 | 1.015, 2.423 | **0.043** |
| Nancy Index | 72 | 1.002 | 0.518, 1.939 | 0.995 |  |  |  |  |
| Platelets | 72 | 0.992 | 0.983, 1.001 | **0.066** | 72 | 0.993 | 0.84, 1.003 | 0.195 |
| Ferritin | 72 | 1.000 | 0.995, 1.005 | 0.878 |  |  |  |  |
|  |  |  |  |  |  |  |  |  |
| Univariate and Multivariable analysis of variables associated with response at 6 months | | | | | | | | |
|  | Univariate | | | | Multivariable | | | |
| Variables at baseline | n | Odds ratio | 95% CI | ***P* value** | n | Odds ratio | 95% CI | ***P* value** |
| Line of therapy | 60 |  |  |  |  |  |  |  |
| ATN |  |  |  |  |  |  |  |  |
| ATE |  | 0.968 | 0.319, 2.940 | 0.955 |  |  |  |  |
| Age (years) | 60 | 1.001 | 0.963, 1.040 | 0.953 |  |  |  |  |
| Sex | 60 |  |  |  |  |  |  |  |
| Female |  |  |  |  |  |  |  |  |
| Male |  | 1.471 |  | 0.496 |  |  |  |  |
| BMI | 60 | 1.014 |  | 0.815 |  |  |  |  |
| Disease duration | 60 | 1.026 |  | 0.477 |  |  |  |  |
| Disease extent | 60 |  |  |  |  |  |  |  |
| E1 |  |  |  |  |  |  |  |  |
| E2 |  | 2.400 |  | 0.286 |  |  |  |  |
| E3 |  | 1.333 |  | 0.706 |  |  |  |  |
| Smoker | 60 |  |  |  |  |  |  |  |
| Never |  |  |  |  |  |  |  |  |
| Former |  | 1.320 |  | 0.660 |  |  |  |  |
| Current |  | 0.440 |  | 0.440 |  |  |  |  |
| EIM | 60 |  |  |  |  |  |  |  |
| No |  |  |  |  |  |  |  |  |
| Yes |  | 0.492 |  | 0.215 |  |  |  |  |
| Concomitant therapy | 60 |  |  |  |  |  |  |  |
| No |  |  |  |  |  |  |  |  |
| 5-ASA |  | 1.729 |  | 0.340 |  |  |  |  |
| Prednisone use | 60 |  |  |  |  |  |  |  |
| No |  |  |  |  |  |  |  |  |
| Yes |  | 1.500 |  | 0.491 |  |  |  |  |
| Budesonide use | 60 |  |  |  |  |  |  |  |
| No |  |  |  |  |  |  |  |  |
| Yes |  | 1.300 | 0.386, 4.380 | 0.672 |  |  |  |  |
| SCCAI | 57 | 1.191 | 0.821, 1.726 | 0.347 |  |  |  |  |
| Nancy Index | 57 | 1.116 | 0.515, 2.415 | 0.778 |  |  |  |  |
| Platelets | 57 | 1.002 | 0.994, 1.010 | 0.634 |  |  |  |  |
| Ferritin | 57 | 1.000 | 0.995, 1.006 | 0.909 |  |  |  |  |
|  |  |  |  |  |  |  |  |  |
| Univariate and Multivariable analysis of variables associated with response at 12 months | | | | | | | | |
|  | Univariate | | | | Multivariable | | | |
| Variables at baseline | n | Odds ratio | 95% CI | ***P* value** | n | Odds ratio | 95% CI | ***P* value** |
| Line of therapy | 36 |  |  |  |  |  |  |  |
| ATN |  |  |  |  |  |  |  |  |
| ATE |  | 2.553 | 0.471, 13.612 | 0.279 |  |  |  |  |
| Age (years) | 36 | 1.014 | 0.960, 1.072 | 0.610 |  |  |  |  |
| Sex | 36 |  |  |  |  |  |  |  |
| Female |  |  |  |  |  |  |  |  |
| Male |  | 1.227 | 0.230, 6.548 | 0.811 |  |  |  |  |
| BMI | 36 | 0.913 | 0.788, 1.057 | 0.223 |  |  |  |  |
| Disease duration | 36 | 1.026 | 0.913, 1.152 | 0.672 |  |  |  |  |
| Disease extent | 36 |  |  |  |  |  |  |  |
| E1 |  |  |  |  |  |  |  |  |
| E2 |  | 0.750 | 0.064, 8.834 | 0.819 |  |  |  |  |
| E3 |  | 1.625 | 0.115, 22.981 | 0.719 |  |  |  |  |
| Smoker | 36 |  |  |  |  |  |  |  |
| Never |  |  |  |  |  |  |  |  |
| Former |  | 1.067 | 0.199, 5.714 | 0.940 |  |  |  |  |
| Current |  | 430793291.43 | 0.00, | 0.999 |  |  |  |  |
| EIM | 36 |  |  |  |  |  |  |  |
| No |  |  |  |  |  |  |  |  |
| Yes |  | 1.244 | 0.235, 6.576 | 0.797 |  |  |  |  |
| Concomitant therapy | 36 |  |  |  | 36 |  |  |  |
| No |  |  |  |  |  |  |  |  |
| 5-ASA |  | 0.118 | 0.012, 1.108 | **0.061** |  | 0.139 | 0.013, 1.438 | 0.098 |
| Prednisone use | 36 |  |  |  |  |  |  |  |
| No |  |  |  |  |  |  |  |  |
| Yes |  | 0.529 | 0.100, 2.811 | 0.455 |  |  |  |  |
| Budesonide use | 36 |  |  |  |  |  |  |  |
| No |  |  |  |  |  |  |  |  |
| Yes |  | 0.952 | 0.153, 5.942 | 0.958 |  |  |  |  |
| SCCAI | 36 | 1.291 | 0.574, 2.906 | 0.504 |  |  |  |  |
| Nancy Index | 36 | 2.755 | 0.785, 9.674 | **0.111** | 36 | 2.577 | 0.683, 9.727 | 0.159 |
| Platelets | 36 | 1.003 | 0.993, 1.013 | 0.542 |  |  |  |  |
| Ferritin | 36 | 1.002 | 0.995, 1.008 | 0.591 |  |  |  |  |

Note: Bold values indicate P < 0.05

*Abbreviations: SCCAI, simple clinical colitis activity index; CI, confidence interval; ATN, advanced therapy-naïve; ATE, advanced therapy-experienced; BMI, body mass index; EIM, extraintestinal manifestation; 5-ASA, 5-aminosalicylic acid*

Supplementary Table 5: Univariate and Multivariable Analysis of Variables associated with filgotinib Discontinuation using Cox Proportional Hazards Regression

| Univariate and Multivariable analysis of variables associated with filgotinib discontinuation - Cox regression | | | | | | | | |
| --- | --- | --- | --- | --- | --- | --- | --- | --- |
|  | Univariate | | | | Multivariable | | | |
| Variables at baseline | n | Hazard ratio | 95% CI | ***P* value** | n | Hazard ratio | 95% CI | ***P* value** |
| Line of therapy | 100 |  |  |  | 94 |  |  |  |
| ATN |  |  |  |  |  |  |  |  |
| ATE |  | 1.676 | 0.861, 3.263 | **0.129** |  | 1.898 | 0.813, 4.433 | 0.139 |
| Age (years) | 100 | 0.982 | 0.959, 1.006 | **0.141** | 94 | 0.998 | 0.971, 1.025 | 0.866 |
| Sex | 100 |  |  |  |  |  |  |  |
| Female |  |  |  |  |  |  |  |  |
| Male |  | 1.227 | 0.637, 2.361 | 0.541 |  |  |  |  |
| BMI | 100 | 1.005 | 0.943, 1.072 | 0.872 |  |  |  |  |
| Disease duration | 100 | 0.95 | 0.901, 1.003 | **0.062** | 94 | 0.915 | 0.845, 0.991 | **0.028** |
| Disease extent | 100 |  |  |  |  |  |  |  |
| E1 |  |  |  |  |  |  |  |  |
| E2 |  | 1.224 | 0.406, 3.690 | 0.719 |  |  |  |  |
| E3 |  | 1.572 | 0.537, 4.601 | 0.409 |  |  |  |  |
| Smoker | 100 |  |  |  |  |  |  |  |
| Never |  |  |  |  |  |  |  |  |
| Former |  | 0.706 | 0.356, 1.399 | 0.318 |  |  |  |  |
| Current |  | 0.458 | 0.062, 3.379 | 0.444 |  |  |  |  |
| EIM | 100 |  |  |  |  |  |  |  |
| No |  |  |  |  |  |  |  |  |
| Yes |  | 0.667 | 0.338, 1.317 | 0.243 |  |  |  |  |
| Concomitant therapy | 100 |  |  |  | 94 |  |  |  |
| No |  |  |  |  |  |  |  |  |
| 5-ASA |  | 0.621 | 0.314, 1.227 | **0.170** |  | 0.682 | 0.286, 1.625 | 0.387 |
| Prednisone use | 100 |  |  |  |  |  |  |  |
| No |  |  |  |  |  |  |  |  |
| Yes |  | 0.891 | 0.463, 1.715 | 0.730 |  |  |  |  |
| Budesonide use | 100 |  |  |  |  |  |  |  |
| No |  |  |  |  |  |  |  |  |
| Yes |  | 0.986 | 0.499, 1.946 | 0.967 |  |  |  |  |
| SCCAI | 94 | 1.008 | 0.867, 1.172 | 0.915 |  |  |  |  |
| Nancy Index | 94 | 1.027 | 0.633, 1.667 | 0.912 |  |  |  |  |
| Platelets | 94 | 1.003 | 0.999, 1.007 | **0.136** | 94 | 1.004 | 1.000, 1.008 | 0.067 |
| Ferritin | 94 | 1.001 | 0.999, 1.004 | 0.228 |  |  |  |  |

Note: Bold values indicate P < 0.05

*Abbreviations: CI, confidence interval; ATN, advanced therapy-naïve; ATE, advanced therapy-experienced; BMI, body mass index; EIM, extraintestinal manifestation; 5-ASA, 5-aminosalicylic acid; SCCAI, simple clinical colitis activity index*

Supplementary Figure 1: Proportion of patients responding with a negative (red), neutral (amber), and positive (green) score for the IBD Control-8 questionnaire at baseline, post-induction, 6 months, and 12 months

Abbreviations: ATN, advanced therapy-naïve; ATE, advanced therapy-experienced; PI, post-induction
